# Ensuring the Stability of the Genome: DNA Damage Checkpoints

**DOI:** 10.1100/tsw.2001.297

**Published:** 2001-11-20

**Authors:** Christine Latif, Susan H. Harvey, Matthew J. O'Connell

**Affiliations:** Trescowthick Research Laboratories, Peter MacCallum Cancer Institute, Locked Bag 1, A'Beckett St., Melbourne, VIC 8006, Australia

**Keywords:** ATM, ATR, cell cycle regulation, checkpoints, Chk1, Chk2, DNA damage, G1/S, G2/M, oncogenesis, p53, signal transduction pathways, tumourigenesis

## Abstract

The cellular response to DNA damage is vital for the cell's ability to maintain genomic integrity. Checkpoint signalling pathways, which induce a cell cycle arrest in response to DNA damage, are an essential component of this process. This is reflected by the functional conservation of these pathways in all eukaryotes from yeast to mammalian cells. This review will examine the cellular response to DNA damage throughout the cell cycle. A key component of the DNA damage response is checkpoint signalling, which monitors the state of the genome prior to DNA replication (G1/S) and chromosome segregation (G2/M). Checkpoint signalling in model systems including mice, Xenopus laevis, Drosophila melanogaster, and the yeasts Saccharomyces cerevisiae and Schizosaccharomyces pombe have been useful in elucidating these pathways in mammalian cells. An examination of this research, with emphasis on the function of checkpoint proteins, their relationship to DNA repair, and their involvement in oncogenesis is undertaken here.

## CHECKPOINTS AND THE RESPONSE TO DNA DAMAGE

The most fundamental function of a cell is ensuring that all progeny receive complete and accurate genetic information. Maintenance of genomic integrity requires the coordination of many events, including DNA synthesis and chromosomal segregation. However, cells exist in an environment rich in exogenous and endogenous DNA damaging agents, not the least of which are by-products of normal cellular metabolism. Therefore, it is vital that cells are able to respond to

[*]Corresponding author. Courier address: Trescowthick Research Laboratories, Peter MacCallum Cancer Institute, St Andrew's Place, East Melbourne, VIC 3002 Australia. Phone: +61 3 9656 1747; Fax: +61 3 9656 1411
©2001 with author.

DNA damage and minimise the transmission of potentially deleterious mutations. DNA damage checkpoints induce cell cycle delays to coordinate progression of the cell cycle with the detection of DNA damage and its subsequent repair. The DNA damage response is multifaceted, requiring both spatial and temporal regulation; damage checkpoints are therefore only part of a global cellular response. The DNA damage response can be triggered at various stages of the cell cycle, and as a consequence, the damage checkpoints are often studied in isolation. As checkpoint dysfunction has been related to tumourigenesis in mammals, a more holistic understanding of the response to DNA damage is required.

This review will examine the current paradigm of the DNA damage checkpoints, the main contributors to the DNA damage response, and the pathways in which they participate. The possibility of interactions between checkpoint signalling and DNA repair pathways will be discussed. Therefore, this review will focus on two seemingly independent checkpoint pathways: the p53-dependent checkpoint, which occurs before DNA replication in G1 and perhaps during S-phase; and the Chk1-dependent pathway, which monitors the completion of DNA replication and acts to halt cells immediately before mitosis in response to DNA damage in G2. Although other DNA damage checkpoints exist, our understanding of them at the molecular level is not as advanced, and will not be considered here.

## APPROACHES TO THE STUDY OF CHECKPOINT SIGNALLING

The study of model systems has been fundamental in furthering our understanding of DNA damage checkpoint signalling and its relationship to tumourigenesis. Hereditary cancer-predisposition syndromes (Table 1), including Li-Fraumeni syndrome, Ataxia telangiectasia (AT), and Nijmegen breakage syndrome (NBS) have been used to dissect the relationship between checkpoints and the genomic instability of tumours. In addition to information gained from the clinical manifestations of these syndromes, examination of cells taken from these patients has allowed checkpoint signalling in response to DNA damage to be examined at the molecular level. These studies have been complemented by investigations of checkpoint signalling in simpler animal model systems such as *Drosophila melanogaster* and *Xenopus laevis*, which allow molecular and biochemical analysis that is not possible in mammalian cells. In addition, knock-out mice and *in vitro* studies of cells derived from these animals have been used as *in vivo* mammalian models of the DNA damage checkpoint signalling pathways.

An extremely powerful tool in the study of the DNA damage checkpoints is the study of the yeasts *Schizosaccharomyces pombe* and *Saccharomyces cerevisiae*. These simple organisms are easily grown and manipulated to allow fine genetic analysis of the interactions between the proteins involved in the DNA damage response. *S. pombe* is a particularly ideal model organism for the study of the cell cycle, as this yeast grows by apical extension and divides by binary fission; therefore, cell length can be used as a marker of the cell's progression through the cell cycle. To date, there has been remarkable conservation in the control of the G2/M transition between *S. pombe* and higher eukaryotic organisms and yeast studies continue to increase our understanding of the controls that underlie mitotic entry (Table 2).

## INITIATING THE PROCESS: THE DETECTION OF DNA DAMAGE

A family of proteins, which have catalytic domains related to the phosphoinositide 3-kinases (PI3-K), have been identified as upstream components of the DNA integrity checkpoints (for a review see[1]). These can be further subdivided into two groups. The first group consists of Rad3 and Mec1 in *S. pombe* and *S. cerevisiae*, respectively, and their vertebrate homologue ATR (ATM and Rad3 related). The second group includes vertebrate ATM (most likely Tel1 in the yeasts).

**TABLE 1**
**Human Diseases Caused by Cell Cycle Gene Deficiencies**

| Disorder | Genes Involved | Comments |
|---|---|---|
| Li-Fraumeni | p53 | • Transcription factor<br>• G1/S checkpoint<br>• Knock-out mouse viable |
| | Chk2 | • Serine/threonine kinase<br>• G1/S checkpoint<br>• Knock-out mouse viable<br>• Possible pseudogenes |
| Nijmegen Breakage Syndrome (NBS) | Nbs1 | • DSB repair protein<br>• Complex with Mre11 & Rad 50<br>• Part of BASC?<br>• Knock-out mouse embryonic lethal |
| Familial Breast Cancer | BRCA1 | • Involved in DNA repair<br>• BRCT domain protein<br>• G2/M checkpoint |
| Ataxia Telangiectasia (AT) | ATM | • PI3-K like kinase<br>• G1 and G2 checkpoints |

**TABLE 2**
**Conserved DNA Damage Checkpoint Genes**

| Functional Class | Mammals | *S. pombe* | *S. cerevisiae* |
|---|---|---|---|
| PI3K-like proteins | ATM | *tel1* | *Tel1* |
| | ATR | *rad3* | *Mec1* |
| | ? | *rad26* | *DDC2/LCD1* |
| CLC proteins | Rad17 | *rad17* | *Rad24* |
| (RF-C-like proteins) | RFC 2-5 | *rfc2-5* | *RFC 2-5* |
| BRCT proteins | BRAC1 | *rhp9* | *RAD9* |
| | ? | *cut5* | *DPB11* |
| CSC proteins | Rad 1 | *rad1* | *Rad17* |
| (PCNA-like proteins) | Rad9 | *rad9* | *Ddc1* |
| | Hus1 | *hus1* | *Mec3* |
| Effector Kinases | Chk1 | *chk1* | *Chk1* |
| | Chk2 | *cds1* | *Rad53* |

The essential nature of these proteins for the DNA damage checkpoint has been shown by mutations inactivating these proteins that abolish checkpoint responses leading to increased DNA damage sensitivity[2,3,4,5,6].

The inherited tumour-predisposition syndrome AT, which is caused by homozygous mutations in the ATM gene[7], has facilitated the analysis of the ATM class of PI3-K-like molecules. The increased tumour frequency and extreme sensitivity to ionising radiation (IR) observed in AT patients can be attributed to defects in a number of DNA damage signalling pathways, including both G1 and G2 checkpoints, stress signalling, and apoptosis[7]. A role for ATM in these defects has been further suggested by ATM knock-out mice, which exhibit phenotypes that mirror the human condition[8,9,10,11]. $ATM^{-/-}$ cells taken from AT patients or knock-out mice, although highly sensitive to IR are only mildly sensitive to other forms of DNA damage[7]. This indicates that ATM plays an important role in the response to IR, but is less critical in response to other forms of DNA damage. In contrast to ATM, ATR knock-out mice exhibit embryonic lethality and have no associated human syndrome[2,12]. This is significant for two reasons. Firstly, it has complicated the study of the role of ATR in the DNA damage response. Secondly, it indicates that although ATM mutations have an important role in tumourigenesis, the viability of ATM knock-out mice and AT patients indicate that ATM is likely to play a lesser role in the overall response to DNA damage than ATR.

The embryonic lethality of the ATR knock-out mouse is not without precedent, as the G2 DNA damage checkpoint has been shown to be essential in early embryogenesis in other systems. This has been well demonstrated by several elegant studies in *Xenopus* oocytes and *D. melanogaster* mutants, in addition to other mouse models, which indicate that the lethal phenotype associated with several G2 checkpoint genes develops during embryogenesis (for examples see[12,13,14,15,16]). Therefore, the lethality associated with ATR deficiency is likely to be related to the absence of embryonic G1 checkpoints, indicating that cell survival following DNA damage is dependent on a functional G2 DNA damage checkpoint. Consistent with this, $ATR^{-/-}$ embryonic stem (ES) cells isolated from knock-out mice die with a mitotic phenotype indicating that ATR deficiency results in abnormalities during mitosis[12]. Furthermore, studies with a dominant-negative form of ATR show that its inactivation impairs checkpoint response and sensitises mammalian cells to all types of DNA damage[3,4].

Work in *S. pombe* has complemented these studies, demonstrating that the ATR homologue, Rad3, is an essential upstream component of the DNA damage checkpoint. The specific inactivation of a conditional allele of *rad3* was used to define a window during which Rad3 activity is absolutely required for the initiation of the DNA damage response and cell survival[5]. Yeast studies, together with the mammalian studies previously described, contribute to an emerging model in which ATM is involved in checkpoint responses to IR, while ATR is involved in the response to all damaging agents[17]. Several lines of evidence, in particular those using *Xenopus* oocyte extracts, support the hypothesis that ATM and ATR signal directly to two serine/threonine kinases: Chk1, and a structurally unrelated kinase, Chk2, which may possess some overlapping functions[18,19]. Through these kinases, signalling from the upstream components of the DNA damage response culminates in a checkpoint arrest.

Other proteins have been hypothesised to act as upstream components of the DNA damage response, the most noteworthy of which may be BRCA1. BRCA1 is a breast and ovarian cancer susceptibility gene. Human and murine cells harbouring mutations in BRCA1 exhibit DNA damage checkpoint defects[20,21]. BRCA1 is also part of a protein complex termed BASC (BRCA1-associated genome surveillance complex) that contains ATM, a double strand break (DSB) repair complex, mismatch repair proteins (Msh2/6 and Mlh2), and the Bloom’s Syndrome helicase (BLM)[22]. This complex contains a conglomerate of proteins that has been proposed to recognise many different aberrant DNA structures and could therefore be involved in transmitting the damage signal to initiate a checkpoint arrest. Currently, this model is somewhat speculative, particularly because several BASC components are also ATM targets; therefore it is unclear if these proteins associate to allow their function as a complex or as a result of an enzymatic interaction. Further work will therefore be required to clarify what role the BASC complex might play in the response to DNA damage.

Despite the hypothesised existence of “sensor” proteins in the DNA damage response, it is unclear what is being “sensed”: Is it DNA lesions *per se* that are detected and lead to checkpoint activation, or do the checkpoints respond to the fact that repair itself is taking place? As several DNA damage checkpoint proteins have roles in S-phase, or are structurally related to DNA replication enzymes, perhaps we could examine their participation in the DNA damage response by making predictions modelled on S-phase functions. Most of the checkpoint genes have been identified using high volume screening techniques in yeast. Why have no sensor proteins been identified in this way? If DNA repair is "sensed", we would anticipate the existence of DNA repair mutants where repair never initiates and, as a consequence, checkpoint activation does not occur. However, screens for such mutants would be challenging, as should these cells be viable, identifying a specific mutant phenotype could be complex. It is also possible that damage sensors may detect elements common to all forms of DNA damage—perhaps ssDNA, or distortions to the double helix. This is not unprecedented. Recently, it has been shown that the XPC-HR23B complex, which participates in nucleotide excision repair (NER), not only recognises DNA lesions in a highly specific manner, but recruits other components of the NER machinery to the site orchestrating the repair process[23]. One other possibility is that the "sensing" pathways overlap, whereby failure of one pathway to detect damage channels damage into an alternative pathway. Should this be true, the sensing pathways will be even more difficult to dissect. As many DNA damaging agents currently used cause diverse forms of damage, this potentially activates multiple pathways. Further effort will be required to clarify if such pathways are an important element of the upstream signalling of the DNA damage response.

## p53, Chk2 AND THE PREREPLICATION (G1/S) DNA CHECKPOINT

At the molecular level, cell cycle delay is accomplished by inhibiting the activity of the cyclin-dependent kinases (CDKs), bound to their cyclin partners, which promote cell cycle transitions. As these complexes accumulate in the cell, they are maintained in an inactive state by the binding of small CDK inhibitor (CKI) proteins, or through inhibitory phosphorylation events at the N-terminus of the CDK[24]. When the cell has satisfied all the criteria for transition into the next cell cycle phase, these complexes are activated by dissociation from their CKI or by dephosphorylation[25,26]. Checkpoints exert their effects by inhibiting this activation.

The function of the G1/S DNA damage pathway is to allow repair to occur before S-phase and hence avoid replicating mutations during DNA synthesis (Fig. 1). The link between checkpoint control and cancer is primarily related to the high frequency with which genomic instability, particularly a loss of G1/S DNA damage checkpoint control, is observed in tumours. p53 is essential for the G1/S DNA damage response and has been implicated in several cellular pathways including angiogenesis, apoptosis, and cell growth arrest (for a recent review see[27,28,29]). Loss of p53 function has been widely implicated in tumourigenesis resulting in genomic instability[30,31,32,33] and has been associated with the tumour syndrome Li-Fraumeni[34,35,36]. p53 has become one of the best studied and arguably the most important protein involved in the G1/S DNA damage checkpoint.

p53 is posttranslationally modified by both phosphorylation and acetylation, which regulate both p53 protein levels and transactivation activity, respectively[37,38,39,40,41]. Normally, p53 is relatively unstable, but following DNA damage, its transient stabilisation is achieved by phosphorylation on serine-20 (S20). This inhibits binding by MDM2, an E3-ubiquitin ligase, which normally binds p53 and targets it for ubiquitin-mediated degradation[42]. Therefore, inhibition of MDM2 binding results in p53 stabilisation, accumulation, and ultimately activation of p53-responsive genes (for a review see[43,44,45]). Among these genes is MDM2 itself, thus ensuring the transient nature of this response[46].

ATM and Chk2 have vital roles in the regulation of p53 during the G1/S DNA damage response. Initial studies indicated that ATM was required for the stabilisation of p53 by phosphorylation at serine-15 (S15)[47]. More recent studies indicate that the Chk2 kinase phosphorylates p53 at S20 and this is the critical event in displacing Mdm2[37,40,41]. In addition to p53, Chk2 has several other cellular targets, including BRCA1[48]. Chk2 has also been

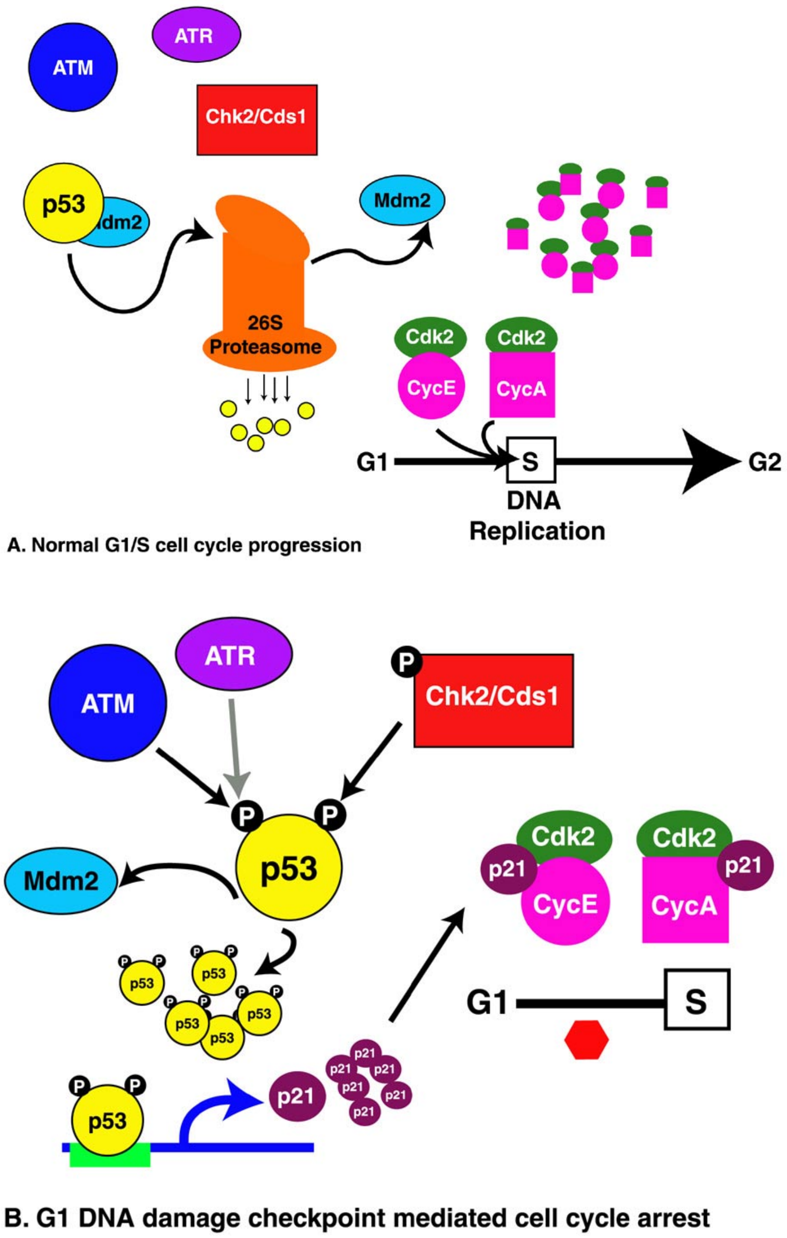

**FIGURE 1**. G1/S cell cycle and checkpoint control. (A) During normal cell cycle progression, the transcription factor, p53, remains unphosphorylated and is targeted to the proteasome for ubiquitin-mediated degradation by Mdm2 binding. As a result, p53 is unable to efficiently activate its target genes. Active cyclinE/Cdk2 and cyclinA/Cdk2 complexes accumulate in the cell, allowing the cell to initiate and complete DNA replication. (B) Following DNA damage, p53 is phosphorylated at serine-15 and serine-20 by ATM and Chk2, respectively. This results in the displacement of Mdm2 and mediates both the stabilisation and accumulation of p53. p53 is therefore free to the activate transcription of its target genes. This includes p21, which accumulates in the cell binding both cyclinE/Cdk2 and cyclinA/Cdk2 complexes, inhibiting them. This cumulates in cell cycle arrest and prevents DNA replication.

identified as a tumour suppressor gene, and in some families where Li-Fraumeni syndrome is not associated with p53 mutation, inherited mutations in Chk2 have been identified[49,50]. However, it must be conceded that these studies have generated some controversy, as their interpretation may be complicated by the presence of several pseudogenes[51]. Nevertheless, Chk2 is involved in the G1/S response to DNA damage. Human Chk2 is phosphorylated by ATM and activated in response to several DNA damage stimuli[12,52,53,54,55,56]. Further, Chk2-deficient murine ES

cells display a modest checkpoint defect in response to IR[40]. Together, these data support a role for ATM signalling to Chk2 in response to DNA damage during S-phase.

The yeast proteins most closely related to Chk2 are Rad53 (*S. cerevisiae*) and Cds1 (*S. pombe*). However, it is unclear to what extent this protein has been conserved through the evolution of these species. Their homology is based on the possession of both kinase and forkhead activation (FHA) domains, but the structural arrangement of these domains differs dramatically between *S. cerevisiae* and other eukaryotes. Specifically, Cds1 and Chk2 have a single FHA domain at their amino terminus and a carboxy-terminal kinase domain. In contrast, Rad53 has FHA domains at both termini and a central kinase domain. Despite these differences, there is some obvious functional homology amongst these proteins as all three have been reported to be involved in S-phase-specific responses to DNA damage[19,41,52,54,55,57,58].

p53-mediated transactivation activity is of particular significance to the G1/S DNA damage response. Following DNA damage, activated p53 is able to promote cell cycle arrest through transactivation of the CDK inhibitor $p21^{cip1/waf1}$[59]. p21 inhibits both cyclinE/Cdk2 and cyclinA/Cdk2 complexes. These complexes are required for entry into and passage through G1 and S-phase, respectively (for a review see[60]). This leads to a cell cycle delay in response to DNA damage[61]. Alternatively, p53 can induce apoptosis when the arrest is insufficient for recovery, for instance, in conditions of high dosage or irreparable DNA damage[62]. Since p53 has various effects on the cell in response to DNA damage, its regulation is another important component of the DNA damage response.

## INHIBITION OF Cdc2 AND THE G2/M DNA DAMAGE CHECKPOINT

Several *S. pombe* proteins have been proposed as playing a very early role in the G2 DNA damage response (Fig. 2). On the basis of molecular modelling and biochemical analysis, three of these proteins—Rad1, Rad9, and Hus1—are hypothesised to form a PCNA-related heterotrimer that could be loaded onto damaged DNA[63,64]. Evidence from other species, including *S. cerevisiae* and mammalian cells, indicates that this sequence of events may be conserved[65,66,67]. Interestingly, the human homologue of Rad1 has been reported to possess an exonuclease activity that could provide a damage processing function to this complex[68]. A fourth protein, Rad17 in *S. pombe*, shares sequence similarity to all five subunits of replication factor-C (RF-C)[69] and interacts with the four smaller RF-C subunits in yeast[70,71]. During DNA replication, the RF-C complex recruits PCNA to form a doughnut-like homotrimer, which acts as a processivity factor to load DNA polymerase δ to its DNA template[64]. An analogous situation has been hypothesised for the action of these proteins. This model predicts that in response to DNA damage, Rad17 binds to its RFC subunits to form a “checkpoint-loading complex” (CLC). It then binds damaged DNA and loads the “checkpoint sliding clamp” (CSC), consisting of Rad1, Rad9, and Hus1 (for a review see[1,72]).

This sequence of events is supported by the analysis of mutants of Rad17 and its RF-C-associated subunits, which have been shown to exhibit checkpoint defects in both yeasts[70,71,73,74,75]. Further evidence of this signalling cascade exists in mammalian cells, where Rad17 is phosphorylated in an ATM/ATR dependent manner in response to DNA damage[76]. Moreover, a Rad17 mutant with altered phosphorylation sites does not associate with the Rad1 component of the CSC and sensitises cells to DNA damage[76]. In *S. pombe*, Rad1 and Rad9 are phosphorylated in a Rad3-dependent manner[65]. Similar signalling from ATM to Rad1 and Rad9 has been identified in mammalian cells, confirming that these proteins are downstream targets of the DNA-damage signal cascade[66,77]. However, it remains unclear whether this process is involved only in triggering the DNA damage response or if it maintains the damage signal until repair is complete.

Another target of the PI3-K-like kinases is the *S. pombe* protein Rad26. Rad26 exists in a complex with Rad3[78] and in response to DNA damage, undergoes a Rad3-dependent phosphor-

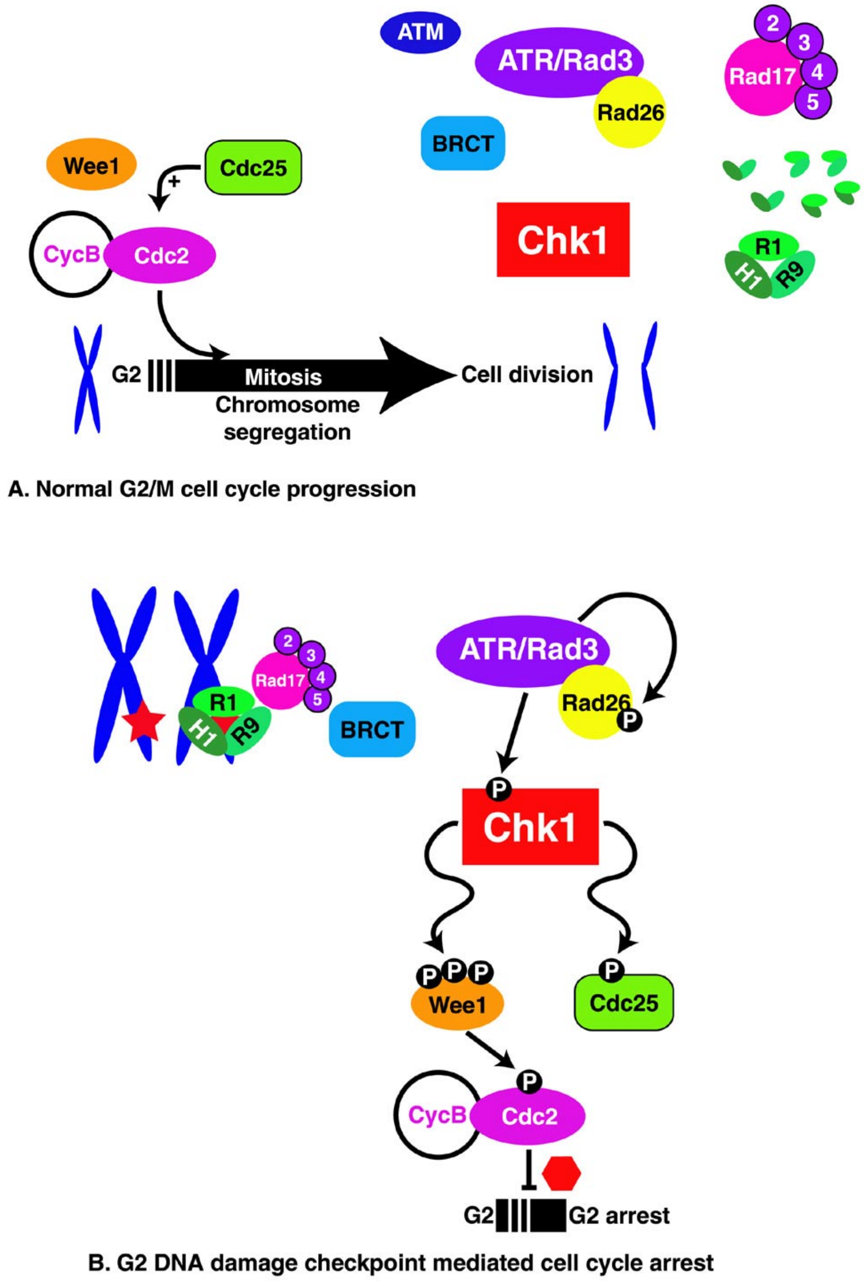

**FIGURE 2**. G2/M cell cycle and checkpoint control. (A) During normal cell cycle progression, cyclin B accumulates within the cell and binds its CDK partner, Cdc2. These complexes are activated by dephosphorylation at tyrosine-15 of Cdc2. This is mediated by the phosphatase Cdc25. These active complexes accumulate, pushing the cell through mitosis and into cytokinesis. Many checkpoint proteins are present in the cell but inactive in the absence of DNA damage. For example, Rad1, Rad9, and Hus1 are present in complexes consisting of various combinations of these proteins. The CSC consisting of a single subunit of Rad1, Rad9, and Hus1, represents only a small proportion of these complexes. (B) Following DNA damage, Rad26 is phosphorylated in a Rad3-dependent manner, which is the earliest known biochemical marker of checkpoint activation in G2. This event has thus far only been described in the yeasts (see text). It has been hypothesised that the CLC consisting of Rad17 and the small subunits of RF-C associate with specific structures in damaged DNA. This may mediate the loading of the CSC, consisting of Rad1, Rad9, and Hus1, onto the damaged site. These events are required for the phosphorylation of Chk1 by ATR/Rad3. Chk1 in turn phosphorylates both Wee1 and Cdc25, leading to the maintenance of tyrosine-15 phosphorylation and therefore inactivation of the cyclinB/Cdc2 complex. This results in an arrest of the cell cycle prior to mitosis.

ylation[78]. This is the earliest known biochemical marker of the G2 DNA damage response described in *S. pombe* cells, indicating that Rad26 plays a central role in the checkpoint. Although functional homologues have been described in *Aspergillus nidulans* (UVSD[79]) and *S. cerevisiae* (Ddc2/Lcd1[80,81]), to date, no structural or functional homologue of Rad26 has been described in mammalian cells. In *S. pombe*, Rad26 is phosphorylated after UV irradiation but not when DNA synthesis is blocked by hydroxyurea (HU) treatment of cells. This phosphorylation is Rad3-dependent but occurs even in the absence of Rad1, Rad9, Rad17, or Hus1[78]. Further, Rad3-dependent phosphorylation of Rad26 that occurs in response to IR does not require the CSC or CLC[78]. However, all other checkpoint signalling events downstream of Rad3 are CSC and CLC dependent. Therefore, it could be hypothesised that Rad3-related checkpoint kinases play a direct role in the recognition of DNA damage only at certain stages of the cell cycle.

In essence, the central target for signalling from upstream checkpoint proteins is the inactivation of the cyclin/CDK complex consisting of Cdc2 and cyclinB. Cdc2 is often described as the universal regulator of mitotic entry because changes in its phosphorylation state have been shown to underlie mitotic onset in all eukaryotic cells (for a review see[82]). The regulation of the cyclinB/Cdc2 complex during DNA damage is somewhat unique. Unlike other cyclin/CDK complexes, cyclinB/Cdc2 is not inhibited by CKIs but is regulated by phosphorylation at tyrosine-15 (Y15) and in higher eukaryotes, also at threonine-14 (T14)[83,84]. During a checkpoint arrest in *S. pombe*, Y15 phosphorylation of cyclinB/Cdc2 is maintained by Chk1's regulation of the kinases Wee1 and Mik1, and the phosphatase Cdc25, which competitively regulate the phosphorylation of cyclinB/Cdc2 at Y15. Similar mechanisms are observed in higher eukaryotes[85,86,87,88,90].

In *S. pombe*, Chk1 is essential for the response to DNA damage[91,92,93]. Similarly, in mammals, the induction of DNA damage in cells where Chk1 function is lacking or impaired results in checkpoint defects[13,15,16,94,95,96,97,98,99]. Further, in *S. pombe*, Chk1p is required for cells to arrest in response to DNA damage, but not the inhibition of DNA replication early in S-phase by HU, demonstrating that cellular responses to DNA damage and faults in DNA replication may be independent and perhaps cell cycle regulated[92,100].

DNA damage results in a phosphorylation of Chk1 only in the presence of Rad3/26 and the CSC and CLC proteins[5,92,93,100]. Several lines of evidence indicate that this signalling is conserved in mammalian cells where Chk1 is phosphorylated by ATR at Serine-315 and Serine-345 in response to UV, HU, and IR[6,18,77,97,101]. Perhaps more intriguingly, DNA damaging agents have been shown to induce phosphorylation of human Chk1, causing a change in its intrinsic kinase activity[102]. Mouse embryos lacking Chk1 exhibit gross nuclear abnormalities resulting in lethality *in vivo* and apoptosis in cultured cells[13], a phenotype strikingly reminiscent of the ATR and Hus1 knock-out mice[2,12]. The strongest evidence linking the function of ATR and Chk1 comes from studies of *Xenopus* egg extracts, where the phosphorylation of Chk1 by ATR is essential for the checkpoint response to unreplicated or UV-damaged DNA[18]. This demonstrates a mechanistic link between the detection of DNA damage and the transduction of signals from upstream checkpoint kinases via Chk1 to the downstream cell-cycle machinery.

Most checkpoint analyses indicate that Chk1 inhibits cylinB/Cdc2 through inactivation of Cdc25. In human cells, there are three isoforms of Cdc25, of which Cdc25B and Cdc25C are thought to be active at G2/M[103,104]. The inhibition of Cdc25 by Chk1 is thought to be mediated by Serine-216 phosphorylation and subsequent binding to 14-3-3 proteins[105,106,107,108,109,110,111]. The significance of 14-3-3 binding is a difficult issue to address. In *S. pombe*, the phosphorylation pattern of Cdc25 and the amount in complex with 14-3-3 proteins is unaltered by DNA damage or by Chk1 deficiency[109,112]. Similarly, immunodepletion of Chk1 from *Xenopus* egg extracts reduces phosphorylation of Cdc25, but by less than 20%[113] and Cdc25 phosphorylation is unaltered by DNA damage[110]. Therefore, although evidence of 14-3-3 binding by both Chk1 and Cdc25

exists[105,106,107,110,112,115] the significance of this interaction for the regulation of the G2 DNA damage checkpoint remains uncertain.

As Chk1 has been shown to phosphorylate human Cdc25C *in vitro*, it has been suggested that this modification occurs during the G2 checkpoint arrest and maintains Y15 phosphorylation of cyclinB/Cdc2 by inactivation of Cdc25C[105,106,107,109,110,111,114,115,116,117]. Cytoplasmic retention of Cdc25, promoted by Chk1-mediated phosphorylation, has also been proposed as an important event in checkpoint signalling[108,111,114]. Consequent to these initial studies performed in *S. pombe*, additional research has transpired that questions the importance of this event in terms of mediating a checkpoint-induced arrest[116]. Although it seems that Cdc25 homologues are legitimate *in vivo* targets of Chk1, more details of precise molecular events are required. For example, current models are difficult to reconcile with reports that show phosphorylation of Cdc25C by Chk1 is not regulated by the cell cycle or even induced by DNA damage[118]. Furthermore, analysis of the murine Cdc25C amino acid sequence reveals that the Ser-216 targeted by Chk1 is not an evolutionarily conserved residue, and the Cdc25C knock-out mouse exhibits no obvious phenotype, including no checkpoint defects[119]. Although this could be taken as evidence that Cdc25C is not a Chk1 target *in vivo*, it is more likely that some functional redundancy between the Cdc25B and Cdc25C homologues exists. Clarification of this issue will await phenotypic analysis of Cdc25B and Cdc25B/C knock-out mice.

A great deal of evidence exists to support a direct link for Chk1's regulation of Cdc2 via Cdc25; however, there is also evidence for signalling by Chk1 to Cdc2 via the Y15 kinases, Wee1 and Mik1. Mik1 is normally expressed only in S-phase, however, some reports indicate that it is upregulated in response to DNA damage[87,88]. Although Mik1 may play a role in checkpoint activation, it appears to have a lesser role than Wee1[87,88,90]. In *S. pombe*, overexpression of Chk1 leads to a Wee1-dependent G2 arrest[86]. Wee1 is a good *in vitro* substrate for Chk1, and both Chk1 overexpression and DNA damage lead to hyperphosphorylation of Wee1, substantially stabilising the protein, and consequently increasing cellular Wee1 kinase activity[86]. These observations are further explained by genetic analysis of cells in which both Cdc25 and Wee1 function are absent. Such cells are both checkpoint defective and hypersensitive to DNA damaging agents[85]. These findings have been supported by parallel studies in *Xenopus* oocyte extracts[120] and *Drosophila*[14] and come together to support a "double-lock" model of checkpoint control, whereby Cdc2 is inhibited by Chk1 signalling through both Wee1 and Cdc25 in response to DNA damage in G2.

As Chk1 plays such a key role in the G2 DNA damage response, its interaction with other proteins is of considerable interest. Claspin has recently been identified as a novel protein in *Xenopus* that binds phosphorylated Chk1[121]. Immunodepletion of claspin lead to reduced Chk1 phosphorylation and activation, resulting in a failure of the DNA replication checkpoint induced by aphidicolin. This indicates that claspin is an important upstream regulator of Chk1[121]. Although a human homologue of claspin has been identified, no yeast equivalent has been found, and the significance of this interaction remains uncertain.

Also noteworthy are reported interactions between Chk1 and Crb2 (also known as Rhp9), the *S. pombe* homologue of *S. cerevisiae* RAD9[122,123]. Crb2 and RAD9 contain BRCT (BRCA1 C-Terminus) domains, a protein-protein interaction domain common to many proteins involved in DNA interactions. Crb2 and RAD9 are involved in genomic integrity and exhibit checkpoint defects in response to DNA damage[122,123,124,125]. In *S. cerevisiae*, RAD9, although not a protein kinase, has been shown to be required for the phosphorylation and activation of RAD53 in response to DNA damage[126,127].

## CHECKPOINT CROSS-TALK: DO INTERACTIONS EXIST BETWEEN THE CHECKPOINTS?

As *S. pombe* cells lacking Chk1 become sensitised to UV irradiation in G2 and late S-phase cells, there is some suggestion that Chk1 may also play a role in the DNA damage response during S-phase in addition to its well-established role through G2/M[91]. In *S. pombe*, Chk1 becomes phosphorylated only in response to DNA damage in the G2 phase of the cell cycle[93]. The singular exception is the HU-induced phosphorylation of Chk1 in the absence of Cds1[57]. Furthermore, when both Chk1 and Cds1 are absent, cells are unable to arrest in response to DNA damage[57]. This is often taken to suggest that Chk1 might have a role in S-phase. However, because Chk1 is not generally phosphorylated in response to replication blocks, it is more likely that in *S. pombe*, its involvement is actually in response to DNA damage. For example, it could be that Cds1 normally stabilises stalled replication forks so they are not detected as DNA damage. In this regard, it is noteworthy that Cds1 was originally isolated as a high-copy suppressor of a temperature-sensitive allele of DNA polymerase α[58]. Moreover, one substrate of Cds1 in *S. pombe* is Hsk1 (Cdc7 in *S. cerevisiae*), which plays an important role in S-phase, specifically the initiation of replication[128,129,130]. As Cdc7-related kinases have been identified in other eukaryotes including *Xenopus*, mice, and humans, this interaction could be conserved in higher eukaryotes[131,132,133,134] and represents an interesting link between the checkpoint response and the replication machinery of the cell.

In mammalian cells, there is some evidence that indicates a role for Chk1 in an early S-phase response. Chk1 is phosphorylated by ATR in response to DNA replication blocks in *Xenopus* egg extracts[18]. Furthermore, in human tissue culture cells, there is some indication that Chk1 might be involved in a rapid p53-independent response to DNA damage during S-phase through regulation of Cdc25A stability[135]. This response is hypothesised to precede a sustained DNA damage response involving p53 during G1/S[60,135,136,137]. Dissection of Chk1 function in mammalian cells will be more complicated than in *S. pombe*. Without cells lacking Chk1 and/or Chk2 it is difficult to clarify what role, if any, Chk1 plays in the DNA damage response at this stage of the cell cycle. This is generally true of all cell-cycle genes and is of concern, since it appears that in mammalian cells particularly, the response of cells to DNA damage may rely implicitly on the position of the cells within the cell cycle.

The existence of other "cross-talk" between the proteins involved in the G1/S and G2/M cell-cycle response to DNA damage has been hypothesised. In particular, several studies have proposed a role for p53 in G2. p53 overexpression or its transactivation of p21 has been hypothesised to induce the down-regulation of cyclin B and result in decreased Cdc2 activity by reducing the pool of active cyclinB/Cdc2 complexes[138,139,140,141,142]. However, an early study showed that p21 is associated with cyclinA/Cdk2 complexes and not cyclinB/Cdc2 complexes *in vivo*[83], indicating that the arrest observed in some studies may actually be a late S-phase arrest.

Studies of p53 function in several mammalian cell lines have failed to resolve what role, if any, p53 plays in the G2 DNA damage response. Some studies indicate that p53-deficient cells can still arrest in response to DNA damage[143,144]; however, cells overexpressing p53 arrest in G2[143,145,146,147,148]. The analysis of overexpression data is always complicated, as the effects on the cell are likely to be partially nonspecific and therefore difficult to interpret. The analysis of the results is complicated further by the use of asynchronous populations of cells or very large doses of IR to induce damage.

Most recently, a study of two matched HCT116-based colon cancer cell lines either wild type or mutant for p53 function, has shown p53 is dispensable for the G2 DNA damage checkpoint when cells are specifically irradiated during G2. Moreover, the upregulation of p53 through serine-20 phosphorylation does not occur in G2[94]. In this study, it is Chk1 activity, not p53 activity, that is essential in the G2 response to DNA damage; interference with Chk1 activity in p53-deficient cells was found to sensitise cells to IR. Furthermore, several novel compounds have been shown to instigate a cell cycle arrest in cells lacking functional p53[96,149,150]. This highlights a new direction for approaches to cancer therapy, which sensitises cancer cells deficient in the G1/S checkpoint to chemotherapeutic agents by abrogating the G2/M checkpoint through Chk1 inhibition.

## ANOTHER LEVEL OF CHECKPOINT REGULATION: LINKS BETWEEN CHECK POINTS AND DNA REPAIR

The idea that maintenance of the checkpoint response is linked to repair and therefore separate from the initiation of the DNA damage checkpoint stems from studies in *S. pombe*. Firstly, a conditional allele of Rad3 has been used to show that Rad3 is required before and at the point of DNA damage, and its inactivation within this time results in a checkpoint defect. However, if Rad3 is inactivated only minutes after irradiation—that is, after the checkpoint is established—there is no effect of the checkpoint proficiency of the cells[5]. This clearly demonstrates Rad3 is involved in the initiation and not the maintenance of the DNA damage response. Secondly, *S. pombe* cells carrying a specific mutation of rad18 (rad18-74), which encodes an essential structural maintenance of chromosome (SMC)-related protein that is also required for DNA repair pathways, do not arrest in response to DNA damage[151]. However, Chk1 is phosphorylated with normal kinetics in these cells, indicating that the checkpoint has been activated[151]. Taken together, these studies indicate that the pathways underlying the initiation and maintenance of the checkpoint in response to DNA damage are partially independent, and it is likely that the maintenance pathway will link to a yet to be identified “repair monitoring system”. The exact nature of this interaction is an obvious area for further research.

Instinctively, we might expect that communication between the repair and checkpoint proteins would exist, allowing the cell to coordinate ongoing repair with its cell-cycle checkpoints. In both yeasts and mammals, a large number of repair genes are transcriptionally regulated by DNA damage[152,153]. For example, p53 is involved in the DNA damage induced regulation of many genes, most recently, it appears to directly contribute to repair by inducing p53R2, a ribonucleotide reductase (RNR) subunit which provides nucleotides which are essential in the repair process[154,155,156].

Moreover, links between DNA repair and the DNA damage checkpoint response have been suggested to act through the protein Nbs1. The Nbs1 protein, mutated in the disorder Nijmegen breakage syndrome, exists in a complex with the human homologues of Mre11 and Rad50[157,158] and is required for both nonhomologous end joining (NHEJ) and homologous recombination of double strand breaks in DNA. In addition, Mre11 is mutated in another AT-like syndrome[159] and AT cells show a reduced capacity to rejoin strand breaks[160]. The Nbs1-Mre11-Rad50 complex is a component of BASC with BRCA1 and ATM, which are required for DNA damage-induced homologous recombination[22,161]. ATM, as part of the DNA damage response, may regulate these repair proteins, although currently this model requires further investigation.

In addition, many repair proteins, including yeast Rad55[162] and RPA[163], are phosphorylated in response to DNA damage. Further, many checkpoint genes in yeast are involved in telomere maintenance that involves some repair-like functions such as recombination[122,164,165]. Could this indicate some repair function in the checkpoint genes themselves? Although ATM and ATR may play a role in the regulation of repair functions, similar roles for other checkpoint genes are yet to be uncovered.

Clearly, there is good evidence of a link between repair and checkpoint function. Further elucidation of these signalling pathways could be greatly assisted by novel technologies such as microarray analysis. Already, one study has used such an approach to examine the effect of IR on gene regulation in a human myeloid leukaemia cell line[166]. Further studies will undoubtedly illuminate the signalling pathways involved in the cellular response to DNA damage and create further links between these pathways and DNA repair.

## SO, WHAT NEXT? USING RESEARCH TO CREATE NOVEL CANCER THERAPEUTICS

Current studies surrounding the DNA damage checkpoint consider the responses independently, but the *in vivo* situation is unlikely to be as simple. When looking at the checkpoints globally, it becomes apparent that far more complexity in the cell cycle exists than is currently appreciated. It is clear that components of the core cell cycle communicate to ensure that cell cycle events occur unidirectionally. It is therefore unlikely that other cell-cycle programs would differ. This leads to further questions: How does the cell decide which damage pathway to activate and how is this decision communicated to the proteins that regulate the cell cycle? Does the cell sense its position in the cell cycle, and if so, how? Are both pathways activated simultaneously? With some consideration it becomes clear that despite many advances in our understanding of the molecular nature of checkpoint signalling, many questions remain unanswered. As the DNA damage response in cells has important consequences in terms of cancer onset, progression, and treatment, a greater understanding of these interactions becomes more imperative. We anticipate that further research will lead not only to an enrichment of our knowledge but advances in our strategies for a cancer therapy.

## NOTE ADDED IN PROOF

Since the submission of this review, it has been demonstrated, using chromatin immunoprecipitations and cell localisation studies, that Mec1 (Rad3/ATR) and the CSC (Rad17, DDC1, and Mec3) are recruited to sites of DNA damage. Furthurmore, this localisation is dependent on components of the CLC (Rad24). These appear to be independent events, suggesting the existence of two mechanisms by which the cell can “sense” DNA damage and signal its presence to the downstream components of the G2 DNA damage checkpoint.[167,168]

## ACKNOWLEDGEMENTS

We thank Drs. Nicole den Elzen, Andrew Cuddihy, and Jeanette Raleigh for critical reading of this manuscript. Research in our laboratory is supported by grants from the Australian Research Council and the National Health and Medical Research Council. C.L. is a recipient of an Australian Postgraduate Award. S.H.H. is a recipient of a Melbourne Research Scholarship. M.O’C. is a Scholar of the Leukaemia and Lymphoma Society.
